# Bulky Isolated Adrenal Metastasis as First Presentation of Occult Hepatocellular Carcinoma (HCC) in a Patient with a Synchronous Squamous Carcinoma of the Tongue

**DOI:** 10.2174/0118715303337748240910093934

**Published:** 2024-10-10

**Authors:** Marco Lodin, Alberto Ragni, Valerio Renzelli, Maura Rossi, Elena Silvia Traverso, Marco Gallo

**Affiliations:** 1 General Surgery Unit, Azienda Ospedaliero-Universitaria SS. Antonio e Biagio e Cesare Arrigo of Alessandria, Alessandria, Italy;; 2 Endocrinology and Metabolic Diseases Unit, Azienda Ospedaliero-Universitaria SS. Antonio e Biagio e Cesare Arrigo of Alessandria, Alessandria, Italy;; 3 Diabetologist and Endocrinologist, Rome, Italy;; 4 Oncology Unit, Azienda Ospedaliero-Universitaria SS. Antonio e Biagio e Cesare Arrigo of Alessandria, Alessandria, Italy

**Keywords:** Isolated adrenal metastasis, occult hepatocellular carcinoma, HCC, bulky lesion, squamous carcinoma, sorafenib, primary cancer

## Abstract

**Background:**

The diagnostic workup of an adrenal mass should always rule out the possibility of an adrenal metastasis, especially in a patient followed-up for a known primitive cancer. Sometimes, however, the incidental finding of a bulky lesion in a cancer patient can lead to the unexpected diagnosis of metastasis from a second occult cancer. Here, we report the case of a voluminous, isolated left adrenal metastasis from unknown and persistently occult hepatocellular carcinoma (HCC), incidentally found during the follow-up for squamous carcinoma of the tongue.

**Case Description:**

A 72-year-old HBV/HCV-negative male patient with a history of alcohol abuse was referred to our hospital for gastric bleeding. Some weeks before, the patient was operated on for a locally advanced squamous cell carcinoma of the tongue, which required cervical lymph node neck dissection, temporary tracheostomy, jejunostomy, and plastic reconstruction. Subsequent diagnostic imaging revealed a left adrenal mass sized 9x15 cm with suspicious features. The hormonal workout was negative for pheochromocytoma and a hyperfunctioning adrenal lesion. The patient underwent laparotomic left adrenalectomy. The exploration of the liver was compatible with alcoholic cirrhosis and did not reveal any other palpable lesion. The adrenal mass histologically turned out to be a poorly differentiated G3 HCC. Subsequent radiological exams were unable to identify the primary liver lesion or any other neoplasms. Conversely, α-FP levels were initially high but reduced after treatment with sorafenib. After 2 years of follow-up, the patient is alive and well, albeit with evidence of locoregional inter-aortocaval lymphadenopathy. The primary HCC has never been identified, thus suggesting the hypothesis of a diffuse cirrhosis-like HCC.

**Conclusion:**

The suspicion of an adrenal metastasis in a patient with primary cancer with a low potential for adrenal metastatic spreading must raise the diagnostic suspect for another synchronous occult cancer beyond that for primary adrenal cancer. HCC can rarely first manifest as a metastatic adrenal lesion.

## 1 INTRODUCTION

### 1.1 Background

The finding of an adrenal lesion in a patient with a known primary cancer is a relatively frequent event, especially for tumours with a high propensity to metastasize to theadrenal gland (*e.g.,* lung, kidney, hepatocellular carcinoma (HCC), and melanoma) [1-3]. The evaluation of an adrenal lesion in this setting requires a thorough clinical, hormonal, and imaging work-up to differentiate metastatic disease from primary adrenal tumours. A biopsy of the adrenal lesion should be evaluated, especially in cases with inconclusive imaging and when histological data are required to direct treatments, even if the risk of adverse events (hemorrhage, neoplastic seeding) must be taken into consideration [4]. Sometimes, especially in the case of a single adrenal lesion, adrenalectomy represents a valid and effective therapeutic option in prolonging overall survival, and this is also the case for HCC [5]. In the follow-up of cancer with a low probability of adrenal metastatic spreading, such as in the case of squamous carcinoma of the tongue, the subsequent finding (sometimes incidental) of an adrenal lesion with malignant imaging characteristics must include in the differential diagnosis not only an adrenocortical carcinoma (ACC) but also, albeit rare, the chance of metastasis from an occult synchronous tumor.

### 1.2 Rationale and Knowledge Gap

HCC is a highly aggressive neoplasm often presenting with locally advanced or metastatic disease. Consequently, despite recent progress and therapeutic improvements, overall survival remains poor. Only a few case reports have described the unusual event of an HCC presenting with an adrenal metastatic lesion [1, 6, 7]. To the best of our knowledge, this is the first case in which the finding of a bulky adrenal metastasis from a persistently occult HCC is combined with a synchronous squamous carcinoma of the tongue.

## 2 CASE PRESENTATION

**Fig. (1):**
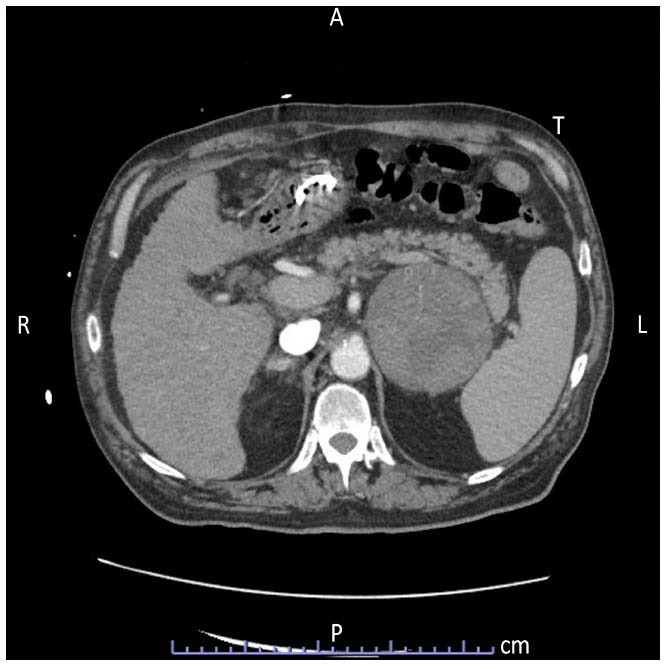
Pre-operative CT showing the left adrenal lesion.

A 72-year-old HBV/HCV-negative male patient with a history of alcohol abuse was referred to our hospital for gastric bleeding. Some weeks before, the patient had undergone right hemi-pelvi-glossectomy with right lymph node neck dissection (I-IV levels), temporary tracheostomy, jejunostomy, and reconstruction with myocutaneous flap of the right pectoralis major muscle for the treatment of an ulcerated squamous cell carcinoma of the middle-distal third of the right lingual border (pT4aN0; Stage IVa according to AJCC 2017, 8^th^ edit.). He underwent a total body CT scan, which highlighted a left adrenal mass sized 9x15 cm with hypodense areas and a necrotic-colliquative density, which imprinted the splenic parenchyma and the splenic vein without infiltration, suspicious for a primitive adrenal malignancy (adrenocortical carcinoma, ACC), or for a metastatic lesion (Fig. **1**). The subsequent ^18^FDG-PET showed a mild and non-homogeneous low signal gradient in the left adrenal gland, some small photopenic areas, as well as necrotic areas (SUV max 3.2). The hormonal workout was negative for pheochromocytoma and for a hyperfunctioning adrenal lesion (Cushing syndrome, primary hyperaldosteronism).

Given the clinical suspicion of a primitive adrenal malignancy, a biopsy of the adrenal lesion was considered but not undertaken due to the potential risk of neoplastic seeding.

**Fig. (2):**
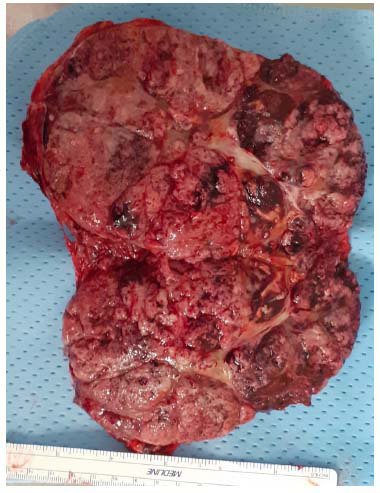
Surgery specimen of the adrenal lesion.

The patient, therefore, underwent laparotomic left adrenalectomy (Fig. **2**). The exploration of the abdominal cavity highlighted the micro-macronodular appearance of the liver compatible with alcoholic cirrhosis and did not reveal any other palpable lesion. The final pathology exam ruled out an ACC, as well as a metastasis from the squamous cell carcinoma of the tongue while orienting toward a poorly differentiated G3 hepatocellular carcinoma (Ki67 50%; IHC staining for chromogranin, synaptophysin, PAX8, CK7, CK20, p16, p63 negative; IHC staining for glypican, hepatocyte, CK AE1-3, AMACR positive at various degrees). Following the histological finding, the patient underwent abdominal ultrasound, CT, and MRI with liver-specific contrast, which were unable to identify the primary liver lesion or any other neoplasms. Conversely, α-FP levels were highly suggestive of an HCC (8,400 ng/mL, normal range 0-7 ng/mL). Suspecting a diffuse cirrhosis-like HCC, the patient was first proposed systemic therapy with atezolizumab/bevacizumab, which was refused. Thereafter, he agreed to be treated with sorafenib and was included in a close clinical and radiological follow-up schedule. Laboratory exams showed a progressive decline in α-FP levels (nadir 169 U/L), but also subsequent diagnostic investigations did not show macroscopic evidence of any liver lesion. At a follow-up of 24 months post-surgery, the patient is alive and well, with ^18^FDG-PET showing extensive inter-aortocaval lymphadenopathy as the only neoplastic localization.

## 3 DISCUSSION

The finding of adrenal metastasis from HCC is an infrequent occurrence in clinical practice [8]. However, adrenal glands have been considered among the most frequent sites of HCC metastases in autoptic studies [9, 10].

Given the high adrenal blood supply and the fact that both adrenal glands seem equally affected, the plausible pathogenetic mechanism is hematogenic spread [11].

Adrenal metastases from HCC are usually synchronous, while metachronous metastases are rare [12, 13] and often occur years after the primary diagnosis [3] or after liver transplant [14].

Our case report represents an even rarer occurrence, an adrenal lesion as the first manifestation of HCC.

Adrenal metastases are usually found in advanced HCC stages, with poor liver function and patient performance status, thus limiting treatment options [1].

In patients with good performance status and clinically fit, surgery is the only curative option for adrenal metastases from HCC. Compared to other therapeutic options, surgery has shown potential survival benefits [15], especially when combined with effective treatment of the primary tumor (*e.g.,* liver transplantation or resection) [5, 16]. When general conditions or extensive metastatic spread do not allow for surgical intervention, minimally invasive/percutaneous procedures (like trans-arterial chemoembolization, radiofrequency ablation, or percutaneous ethanol injection) may be evaluated for symptoms palliation [1, 17, 18]. These treatment modalities have shown, especially when ablative and embolization techniques are combined [18], a good degree of local tumor control [17], but their feasibility needs to be adequately discussed and planned for in a multidisciplinary tumor board, given their potential limitations. For instance, trans-arterial chemoembolization could be technically arduous to perform due to the complexity of adrenal vasculature (with up to three feeding arteries) [19], while the use of ablation techniques carries the risk of tumor seeding [20].

In our patient, the primary lesion still remains unknown, even two years after the initial diagnosis. To our knowledge, only five cases with an unknown primary tumor have been published to date [1, 21-24] (Table **1**). Our patient shares some features with these reported cases, such as contralateral metastasis and negativity for HBV/HCV viruses. However, upon reviewing the clinical data from those cases, no other significant pathognomonic characteristics could be identified. We found no previously reported cases with a history of head and neck cancer similar to our patient.

Given the hypothesis of diffuse cancer-cirrhosis in our patient, sorafenib therapy was initiated, resulting in a sustained clinical response. This is a rare variant of HCC, with a few previously reported cases in which HCC produces diffuse small nodules mimicking cirrhosis [25, 26].

Regarding the response to sorafenib, we found a case report of a patient treated with sorafenib who developed left adrenal metachronous metastasis, which responded to subsequent treatment with atezolizumab plus bevacizumab [27]. This treatment might be considered in case of disease progression.

## CONCLUSION

In the follow-up of a known malignant neoplasm, particularly one with a low potential for adrenal metastatic spreading, the discovery of an adrenal mass that exhibits a malignant appearance on radiographic imaging warrants thorough consideration. It is essential to include the possibility of an ACC in the differential diagnosis, given the aggressive nature of this tumor. Additionally, the adrenal mass could represent a metastasis from another synchronous occult cancer that may not have been previously detected. In rare instances, HCC can present initially as a metastatic lesion in the adrenal gland, even when it has not been identified in the liver or other typical sites through traditional imaging methods. This scenario poses a diagnostic challenge because HCC might not always be evident through standard imaging techniques, potentially leading to its initial detection as an adrenal mass.

In such cases, measuring serum alpha-fetoprotein (αFP) levels could be a useful diagnostic tool as its detection in the presence of an adrenal mass might support the suspicion of HCC as the underlying primary malignancy. Despite all investigations, sometimes, as in our case, the primary tumor may remain unknown, and systemic treatment might still represent a therapeutic option.

## Figures and Tables

**Table 1:** HCC metastasis without primary known tumor.

Sex	History	Lesion
71y Male [17]	Known unchanged non-PET avid 2.2 cm hepatic lesion and normal AFP levels, considered heterotopic tissue	RA 9.1 x 8.2 × 8.6 cm
69y Male [18]	Left-sided abdominal pain; no known liver disease;	LA 7.6 x 6.9 x 7 cm; no hepatic lesions found
32y Male [19]	Weight loss and abdominal bloating in the last 4 months; acute abdominal pain, hematemesis and melena; HBV+	RA 10 x 7 cm; LA 14 x 12 cm; atrophy of hepatic left lobe and hypertrophy of the caudate, with regenerative nodules; no signs of HCC
76y Male [1]	Macroscopic hematuria and lumbar pain; HBV+;	LA 11.5 x 7.5 cm; no hepatic lesions; 7 months later developed multiple hepatic lesions
44y Female [20]	Epigastric pain, no known liver disease	RA (size not reported) with multiple smaller hepatic lesions interpreted as metastasis

**Abbreviations:** LA: left adrenal gland; RA: right adrenal gland; LT: liver transplant; RT: radiotherapy.

## Data Availability

All data generated or analysed during this study are included in this published article.

## LIST OF ABBREVIATIONS

HCC: Hepatocellular Carcinoma
ACC: Adrenocortical Carcinoma
αFP: Alpha-Fetoprotein

## STANDARDS OF REPORTING

CARE guidelines were followed.
